# FPCAM: A Weighted Dictionary-Driven Model for Single-Cell Annotation in Pulmonary Fibrosis

**DOI:** 10.3390/biology14050479

**Published:** 2025-04-26

**Authors:** Guojun Liu, Yan Shi, Hongxu Huang, Ningkun Xiao, Chuncheng Liu, Hongyu Zhao, Yongqiang Xing, Lu Cai

**Affiliations:** 1 School of Life Science and Technology, Inner Mongolia University of Science and Technology, Baotou 014000, China; gjliu77@gmail.com (G.L.);; 2 Inner Mongolia Key Laboratory of Life Health and Bioinformatics, Inner Mongolia University of Science and Technology, Baotou 014000, China; 3 Department of Immunochemistry, Institution of Chemical Engineering, Ural Federal University, Yekaterinburg 620000, Russia

**Keywords:** cell annotation, scRNA-seq, FPCAM, cell–gene association dictionary

## Abstract

The groundbreaking development of scRNA-seq has significantly improved cellular resolution. However, accurate cell-type annotation remains a major challenge. Existing annotation tools are often limited by their reliance on reference datasets, the heterogeneity of marker genes, and subjective biases introduced through manual intervention, all of which impact annotation accuracy and reliability. To address these limitations, we developed FPCAM, a fully automated pulmonary fibrosis cell-type annotation model. Built on the R Shiny platform, FPCAM utilizes a matrix of up-regulated marker genes and a manually curated gene–cell association dictionary specific to pulmonary fibrosis. It achieves accurate and efficient cell-type annotation through similarity matrix construction and optimized matching algorithms. To evaluate its performance, we compared FPCAM with state-of-the-art annotation models, including SCSA, SingleR, and SciBet. The results showed that FPCAM and SCSA both achieved an accuracy of 89.7%, outperforming SingleR and SciBet. Furthermore, FPCAM demonstrated high accuracy in annotating the external validation dataset GSE135893, successfully identifying multiple cell subtypes. In summary, FPCAM provides an efficient, flexible, and accurate solution for cell-type identification and serves as a powerful tool for scRNA-seq research in pulmonary fibrosis and other related diseases.

## Simple Summary

Identifying which cell types are involved in disease is crucial for developing better treatments. New single-cell RNA sequencing technologies allow researchers to study individual cells in great detail, but accurately labeling these cells remains difficult. Many current tools rely on prior knowledge, which can lead to errors. We developed FPCAM, a fully automated tool that accurately identifies lung-related cell types, especially in diseases like pulmonary fibrosis. FPCAM uses marker gene patterns and a curated reference to make reliable predictions. It outperformed several popular tools and accurately identified specific cell subtypes in a separate dataset. FPCAM is freely available and easy to access online.

## 1. Introduction

Traditional bulk sequencing methods analyze RNA by pooling all the cells from a sample into one collective, which, while providing overall information about the cell population, fails to reveal the heterogeneity between individual cells. This approach often overlooks rare cell types or cells in transitional states [1]. This limitation frequently leads to an insufficient understanding of cell types, functional states, and their interrelationships. In contrast, single-cell RNA sequencing (scRNA-seq) can analyze the gene expression of each individual cell, offering researchers critical insights into cell types, differentiation trajectories, and functional heterogeneity [2]. As a major technological breakthrough in biological research, scRNA-seq allows for the precise resolution of gene expression at the single-cell level, providing a new perspective for understanding the structure and function of complex biological systems [3]. The advent of this technology has enabled researchers to study cellular differences at a higher resolution, identify previously unnoticed rare cell subpopulations, and unveil cellular heterogeneity and dynamic changes in biological processes. This has provided revolutionary insights, especially in fields like cancer, developmental biology, and immunology [4]. Through the high-precision analysis enabled by single-cell sequencing technology, studies not only reveal the diversity, functional networks, and dynamic regulatory mechanisms of lung cells but also construct a multidimensional map that bridges the cognitive gaps of traditional bulk sequencing, laying a solid molecular foundation for targeted treatments and regenerative medicine for lung diseases in the era of precision medicine.

Sikkema et al. constructed the Human Lung Cell Atlas (HLCA), integrating data from 49 datasets to generate a reference atlas containing 2.4 million cells, encompassing all major lung scRNA-seq studies published to date [5]. Madissoon et al. integrated scRNA-seq with spatial transcriptomic data, providing a fine resolution of 80 cell types/states in the human lung and airway [6]. Sun Xin et al. used single-cell RNA sequencing and genetic lineage tracing to reveal newly discovered lung cell types and their rich functional characteristics. They synthesized these data into a comprehensive and practical lung cell census, capturing the identity of cell types in the normal lung and describing their functions, markers, developmental lineage, heterogeneity, regenerative potential, disease associations, and key experimental tools [7]. Travaglini et al. sequenced cells from approximately 75,000 human lung tissues and circulating blood and created a comprehensive HLCA by combining multiple cell annotation methods. This allowed them to define the gene expression profiles and anatomical locations of 58 cell clusters, including 41 of 45 known cell types and 14 novel cell types [8]. Cohen et al. combined tissue developmental dynamics from single-cell RNA-seq analysis with a broad screening of ligand–receptor interactions between cell types, generating a comprehensive cell network for all lung types [9]. Despite groundbreaking advances in single-cell RNA sequencing technology that have provided unprecedented resolution for cell annotation, the efficiency and accuracy of cell-type annotation still face many challenges. Current automated annotation tools rely on reference datasets, the diversity of marker genes, and subjective biases introduced by manual intervention, making it difficult to accurately annotate complex cell subpopulations and cross-platform datasets.

Cell-type annotation is a fundamental and indispensable step in interpreting scRNA-seq data. This process typically involves two main approaches: manual annotation and automated annotation. Automated annotation generally follows two strategies: one classifies cells based on the expression patterns of cell-type-specific marker genes [10,11], while the other leverages machine learning algorithms to transfer cell-type labels from reference datasets to the target dataset [12,13,14,15]. Automated methods offer significant advantages when a reliable library of marker genes is available [16] and high-quality reference datasets can be obtained. These methods can efficiently and reproducibly assign cell-type labels to individual cells or clusters, making them widely used for the initial annotation of scRNA-seq data [17]. Nevertheless, they still face challenges in distinguishing highly similar subpopulations, such as transitional cells along developmental trajectories, or in performing deep annotations of functionally specific subtypes within the immune system.

Among automated strategies, several supervised learning-based methods have demonstrated strong performance in recent years. Tools such as CellTypist, scPred, and ACTINN apply various machine learning models—including logistic regression, support vector machines, and neural networks—to learn from well-annotated reference datasets and accurately predict cell types in new data. CellTypist uses a machine learning approach to classify cells based on the expression patterns of cell-type-specific marker genes, providing accurate predictions by leveraging a reference dataset of well-annotated cell types [18]. scPred employs support vector machines to transfer cell-type labels from a reference dataset to a target dataset, making it effective for reliable annotations in complex scRNA-seq data [15]. ACTINN utilizes deep learning techniques, specifically neural networks, to predict cell types by learning from annotated reference datasets, excelling in handling large and complex scRNA-seq datasets [19]. These methods have gained popularity due to their scalability, reproducibility, and capacity to handle large and complex datasets. However, these methods are heavily dependent on the quality and completeness of the reference datasets, which may lead to inaccurate annotations if rare or uncharacterized cell types are not well represented. Additionally, while they perform well on large datasets, they may struggle with distinguishing cell subtypes or highly similar cell populations, leading to potential misclassifications.

In contrast, expert manual annotation remains widely regarded as the gold standard for cell-type identification [16]. Manual approaches enable researchers to (1) dynamically assess the spatial expression patterns of marker genes to infer cell function; (2) identify novel or rare cell states, especially within ambiguous clusters; and (3) integrate multi-omics evidence to elucidate molecular regulatory networks. However, this process is time-consuming, highly dependent on expert knowledge, and constrained by the completeness and accuracy of current biological databases, such as marker gene repositories. To our knowledge, regardless of the algorithm used, very few are capable of accurately annotating fine-grained cell subtypes. Moreover, their performance in annotating cells associated with lung-related diseases—such as pulmonary fibrosis—is generally limited. Therefore, efficient and accurate annotation remains a major challenge.

This study developed the Fibrosis Pulmonary Cell Type Annotation Model (FPCAM), a comprehensive single-cell annotation platform based on the R Shiny framework. The core innovation lies in the introduction of a weighted voting integration algorithm and a dynamically expandable marker gene library, effectively addressing the annotation ambiguity caused by reference files in existing tools for pulmonary fibrosis disease samples. To evaluate the platform’s performance, we compared it with three mainstream annotation tools: SCSA [20], which is based on hypergeometric testing; SingleR [12], which is driven by reference datasets; and SciBet [21], a probabilistic generative model. A systematic evaluation was conducted using different reference datasets. The results showed that both FPCAM and SCSA achieved an accuracy of 89.7% on our dataset, significantly outperforming SingleR and SciBet. Moreover, SingleR and SciBet exhibited accuracy variation ranges of 0.241 and 0.242, respectively, indicating a strong dependency on reference files during the annotation process. In contrast, FPCAM and SCSA demonstrated higher accuracy and greater stability, making them more reliable solutions for single-cell annotation in pulmonary fibrosis research.

## 2. Materials and Methods

### 2.1. Data Sources

We selected male C57BL/6 mice as the experimental model. The experimental design included two main groups: the control group and the dust-exposure group. Mice in the control group were not subjected to any treatment throughout the experiment, while mice in the dust-exposure group were exposed to silica dust for 4 h per day, continuously for 128 days, simulating the physiological changes associated with long-term exposure to a silica dust environment and the observation of potential lung damage. After the experiment, euthanasia was performed using cervical dislocation. Lung tissue samples were immediately collected through abdominal dissection. During sampling, the lung tissues were first washed with precooled phosphate-buffered saline to remove residual blood and impurities, ensuring sample purity. The cleaned lung tissue samples were quickly frozen and immersed in liquid nitrogen for storage, ensuring tissue integrity and RNA stability. All collected lung tissue samples were labeled and grouped by number, with three independent biological replicates per group to ensure the reliability and statistical significance of the experimental results. All samples were subsequently sent to BGI (www.bgi.com, accessed on 18 August 2024) for next-generation single-cell RNA sequencing. The research protocol was reviewed and approved by the Biomedical Ethics and Biosafety Committee of Inner Mongolia University of Science and Technology. The committee confirmed that all biological experimental procedures and related materials in this study comply with laboratory safety management regulations. The School of Life Science and Technology at Inner Mongolia University of Science and Technology provided the necessary experimental facilities to ensure the safe and effective implementation of the project. All methods were carried out in accordance with relevant guidelines and regulations.

### 2.2. Data Preprocessing and Quality Control

After sequencing, reads can be categorized into different components based on their functions and sources: barcode sequences for cell identification, unique molecular identifier (UMI) sequences for gene expression analysis, and sample insert sequences (RNA reads). Following initial data processing, we utilized STAR alignment software to map RNA reads to the reference genome and calculated the percentage of reads aligned to various genomic regions. By assessing the number of reads mapped to the genome and the abundance of UMIs, we determined the expression levels of individual genes in each cell [22,23,24,25,26,27]. Subsequently, we performed clustering analysis using UMAP. Finally, the FindAllMarkers function in Seurat (parameters: only.pos = TRUE, min.pct = 0.1, test.use = wilcox, logfc.threshold = 0.25, screening parameter: *p*.adjust = 0.05) was employed to compare gene expression between clusters, enabling the identification of marker genes specific to each cluster [28].

### 2.3. Construction of the Pulmonary Fibrosis Annotation Dictionary

Based on a review of the existing scientific literature and ranked according to the importance of the studies, we collected data from six pulmonary fibrosis-related papers by researchers such as Sikkema, Madissoon, Sun, and others. Cell subtypes were classified according to four levels: cell function, cell lineage, cell type, and cell subtype. Based on the frequency of use of the marker genes, we selected the top 10 marker genes using a voting system and calculated the weighted score for each gene. After removing duplicate entries, we identified 627 unique marker genes, which corresponded to 75 distinct cell subtypes. Subsequently, a dictionary-based voting matrix was constructed, with marker genes as rows and cell types as columns. Each element denotes the vote count of a specific marker gene for a given cell type, and the number of marker genes *i* varies across different cell types.

(1) $$V=\left(\begin{array}{cccc} v_{11} & v_{12} & \cdots & v_{1n}\\ v_{21} & v_{22} & \cdots & v_{2n}\\ \vdots & \vdots & \ddots & \vdots \\ v_{m1} & v_{m2} & \cdots & v_{mn} \end{array}\right)$$

Finally, a pulmonary fibrosis gene–cell dictionary was constructed, which was then used as a custom database for subsequent cell-type annotation. This process determines the weight of each marker gene. *Vₘ* represents the “vote count” or weight value of each marker gene in cell type *m*, which is obtained from the “percent” column of the dictionary. *Vₘₙ* denotes the expression weight of a marker gene in each cluster, while *Mₘₙ* represents the weight of marker genes for cell type *m* in cluster *n*. The output weight *Mₘₙ* indicates the contribution of each cell type within a specific cluster. Marker genes with higher weights better represent their corresponding cell types, enhancing the accuracy of cell annotation.

(2) $$M_{mn}=\frac{V_{m}}{\sum_{n}V_{mn}}$$

### 2.4. Construction of the Marker Gene Expression Matrix

First, an expression matrix was generated from scRNA-seq analysis, with each column representing a cell cluster and each row corresponding to a marker gene. Each element in the matrix represents the expression level of a specific marker gene in a given cell cluster, and the number of marker genes *i* varies across different cell clusters.

(3) $$A=\left(\begin{array}{cccc} a_{11} & a_{12} & \ldots & a_{1j}\\ a_{21} & a_{22} & \ldots & a_{2j}\\ \vdots & \vdots & \ddots & \vdots \\ a_{i1} & a_{i2} & \ldots & a_{ij} \end{array}\right)$$

Then, the proportion of each gene in every cell cluster within the expression matrix was calculated by dividing the expression value of each gene by the total expression value of all genes in its respective cell cluster.

(4) $$E_{ij}=\frac{A_{ij}}{\sum_{j}A_{ij}}$$

The relative expression proportion of gene *i* in cluster *j* was calculated by taking *Aᵢⱼ* (the expression value of gene *i* in cluster *j*) and dividing it by the total expression value of that gene across all clusters (summed over all *j*). This allowed for the comparison of different genes’ contributions within the same cluster. Additionally, the weight of each marker gene in a given cell type was determined by dividing its count by the total count of all marker genes within that cell type.

### 2.5. Construction of the Similarity Score Matrix

Finally, each proportion value obtained from the expression matrix is multiplied by the corresponding weight computed from the dictionary voting matrix to generate the similarity score matrix.

(5) $$S_{im}=\left\{\begin{array}{c} \sum_{jn}E_{ij}\times M_{mn}\;,\;if\;genes\;in\;E_{ij}\;and\;M_{mn}\;match\\ 0\;,\;otherwise \end{array}\right.\;$$

This similarity score is calculated based on marker genes and gene expression proportions. By multiplying each gene expression value *Eᵢⱼ* by the marker gene weight *Mₘₙ* and summing over all relevant genes, we obtain the similarity score between a cluster and a specific cell type. These scores help determine the association between each cluster and a known cell type. Through the analysis of the similarity matrix, the most probable cell type for each cluster can be identified. This is crucial for cell annotation, as it enables the identification of the biological characteristics of different cell populations. First, for each cell cluster *i*, we find the maximum similarity score among all cell types *m*, which is given by

(6) $$S_{i}=\arg\underset{m}{\max}\left\{S_{im}\right\}$$

By calculating the difference between the maximum similarity score (*Sₘₐₓ*) and the median similarity score (*S_median_*), we determine the first threshold, *λ*_1_. If the difference between *Sₘₐₓ* and the second-highest similarity score (*Sₘₐₓ* − *S_median_*) exceeds 0.00001, then a second threshold, *λ*_2_, is introduced.

(7) $$\lambda_{1}=S_{max}-S_{median}$$

(8) $$\lambda_{2}=S_{sec-max}-S_{median}$$

Finally, cells are annotated based on the cell type corresponding to the threshold *λ*_1_. If a secondary threshold *λ*_2_ exists, an additional annotation is assigned based on the second-highest similarity score. It is important to note that if the *λ* value is zero or negative, it becomes meaningless and should be disregarded.

### 2.6. Model Evaluation and Comparison

To evaluate the annotation capability of the model, we used accuracy (*Acc*) to measure the matching degree between the predicted cell types and the true labeled cell types. The formula is as follows:

(9) $$Acc=\frac{TP}{TP+FP+FN}$$

The *TP*, *FP*, and *FN* refer to True Positives, False Positives, and False Negatives, respectively. We calculated the accuracy of SCSA, SingleR, SciBet, and FPCAM, statistically analyzing and comparing the predictions of each annotation model. This allowed us to determine the optimal annotation reference dataset and the most effective prediction model. To evaluate the accuracy and consistency of these annotation results, we employed the Boyer–Moore majority voting algorithm for integration. For each cell cluster, the annotation with the highest vote among the six annotation results was considered the final annotation of the reference dataset. During the evaluation, if an annotation result matched the reference dataset exactly, it was awarded one point. Additionally, to assess the variability of different annotation models (e.g., SCSA, SingleR, and SciBet), we calculated their range (*R*):

(10) $$R=|{Acc}_{1}-{Acc}_{2}|$$

The terms *Acc*_1_ and *Acc*_2_ refer to the accuracy of a model based on annotation file 1 and annotation file 2, respectively. To ensure the accuracy and reliability of the cell annotation tools, we selected three commonly used tools for comparative analysis, including SCSA, SingleR, and SciBet. We performed a comprehensive comparison of the performance of these tools with our self-developed tool, using the same input files and specific reference datasets for annotation to ensure a fair comparison. To evaluate the dependency of SCSA, SingleR, and SciBet on annotation files, we used different annotation datasets for comparison. Specifically, SingleR utilized two reference datasets—ImmGen database data and mouse RNA sequencing data—both of which contain extensive immune-cell-type information and mouse RNA sequencing data, offering comprehensive cell-type annotations. SciBet used two datasets: the Tabula Muris (mouse cell atlas), based on single-cell transcriptomic analysis of 20 mouse organs, and lung tissue Smart-seq2 data as the training set. These datasets cover single-cell transcriptomic data from various mouse tissues, providing high diversity and representativeness. To run the SCSA model, we first downloaded marker databases from CellMarker2.0 (http://www.bio-bigdata.center/, accessed on 18 August 2024) and PanglaoDB (https://panglaodb.se/, accessed on 18 August 2024). Then, we defined a custom marker database in table format with two columns: the first column for cell names and the second for gene names. This custom table was subsequently used as the input file for the SCSA model.

### 2.7. Online Data Analysis Platform and Validation Dataset

R Shiny is a framework for building interactive web applications, allowing users to easily integrate data analysis functions with web-based user interfaces. This enables dynamic and interactive data visualization and application demonstrations [29]. We developed the FPCAM online data analysis platform based on R Shiny, which currently supports several features including cell annotation analysis, UMAP visualization, a scoring heatmap generated using the plotScoreHeatmap function from SingleR, and Delta distribution plots using the plotDeltaDistribution function from SingleR. These features enable users to efficiently analyze single-cell transcriptomic data related to pulmonary fibrosis, providing strong support for investigating the roles of different cell types in the disease and their potential clinical implications. We downloaded the publicly available dataset GSE135893 from the GEO (https://www.ncbi.nlm.nih.gov/geo/, accessed on 15 April 2012) database and used it to validate the performance of FPCAM.

## 3. Results

### 3.1. Quantitative Statistics of Sequencing Results

**Figure 1:**
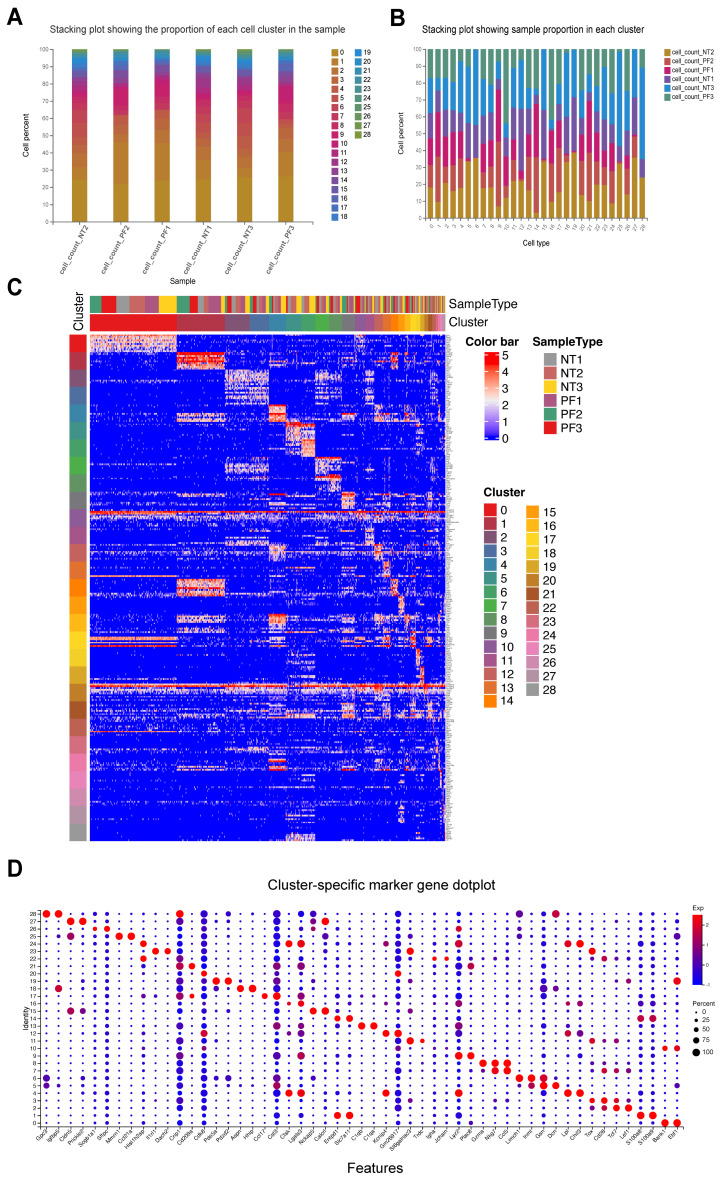
Statistics of cell clusters and their marker genes. (**A**). Stacking plot showing the proportion of each cell cluster in the sample. Horizontal axis is the sample name and vertical axis is the percentage of the cell in each cell type. (**B**)**.** Stacking plot showing sample proportion in each cluster. Horizontal axis is the cell type and vertical axis is the percentage of the cell in each sample. (**C**) Sample-specific marker gene heatmap. An expression heatmap was drawn for the top 10 most significant marker genes in a cluster (avg_logFC ranks the top 10 genes). Each column of the heatmap area represents a cell, and each row represents a gene. (**D**) The two most significant marker genes in each cluster were selected for display in the dot plot. The brighter the color, the higher the expression; the larger the bubble, the higher percentage of the gene.

The basic statistics of the sequencing data are presented in Table S1. Following initial data processing, we utilized STAR alignment software to determine the percentage of reads mapped to different genomic regions (Table S2). Based on the number of reads aligned to the genome and the corresponding UMIs, we quantified the expression levels of each gene in individual cells. These expression levels were then used to derive the fundamental statistical metrics, as summarized in Table S3. A stacking plot illustrates the proportion of each cell cluster identified by UMAP within the sample (Figure 1A). Similarly, another stacking plot depicts the sample composition within each cluster (Figure 1B). We generated a heatmap showcasing the expression patterns of the top 10 most significant marker genes in each cluster, ranked by “avg_logFC”. In the heatmap, each column represents a cell, while each row corresponds to a gene (Figure 1C). Additionally, the two most significant marker genes from each cluster were selected for visualization in a dot plot (Figure 1D). The marker genes and their expression levels across different clusters are available in File S1. The number of up-regulated marker genes across all sample clusters was quantified and is presented in Figure S1.

### 3.2. FPCAM Workflow and Construction of a Pulmonary Fibrosis-Specific Cell–Gene Dictionary

**Figure 2:**
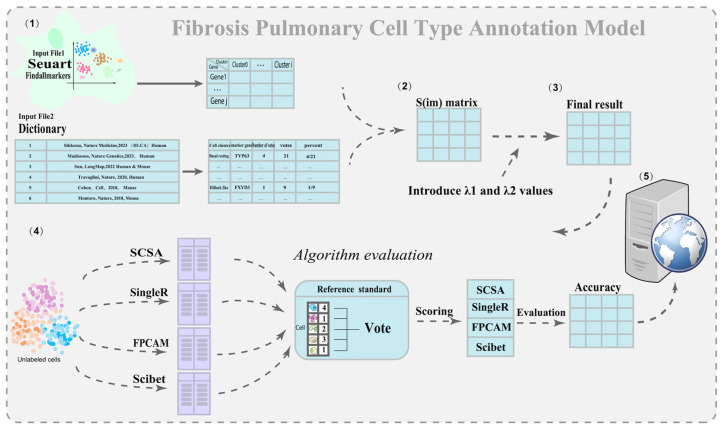
Flowchart of the FPCAM algorithm.

As shown in Figure 2, the technical workflow of this study consists of five main parts: (1) preparing the marker gene expression matrix and constructing a pulmonary fibrosis-specific cell–gene association dictionary matrix; (2) calculating the similarity matrix; (3) introducing an optimal threshold λ, and building a weighted scoring matrix to optimize cell-type annotation; (4) comparing the results with three algorithms and computing the Acc value; (5) developing the FPCAM online web application and conducting downstream bioinformatics analysis. As shown in Table 1, by compiling data from six studies, we identified a total of 627 unique marker genes after removing duplicates. These genes cover 75 cell subtypes, with the number of cell types reported in the literature ranging from 7 to 61. Secondly, a dictionary file needs to be constructed. Table 2 presents the voting count, total votes, and voting ratio of the top 10 marker genes in basal resting cells. For example, the TP63 gene received 4 votes out of a total of 21, resulting in a voting ratio of 4/21. The complete dictionary file can be found in File S2, which serves as a fundamental resource for subsequent data processing and visualization.

### 3.3. Similarity Score Calculation Between Known Cells and Unknown Cell Clusters

**Figure 3:**
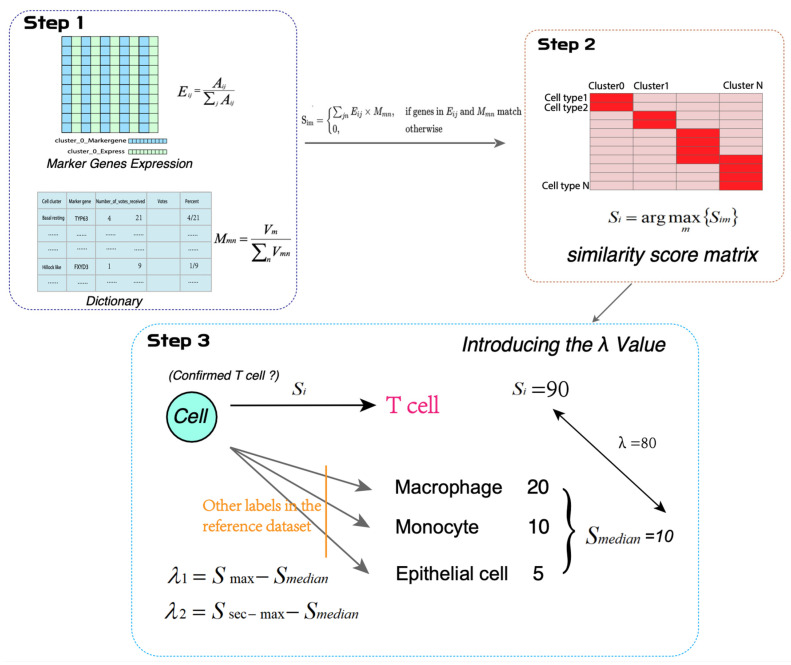
Calculation process of similarity scores between known cells and unknown cell clusters.

FPCAM first addresses issues related to gene expression data and gene weighting in the dictionary. The raw data are processed using the FindAllMarkers function in Seurat, generating a marker gene expression matrix with a gene–cluster binary structure [28]. This matrix is then integrated with a custom dictionary file compiled from sources in the literature. Through the use of Formulas (2), (4), and (5), a similarity matrix is calculated, and the maximum similarity score for each cluster is determined based on Formula (6). These scores reflect the degree of similarity between known cells and unknown clusters. In the process of introducing the optimal threshold λ, the cell-type assignment is mainly based on the λ₁ value, meaning the cell type corresponding to the highest weighted score is considered the preliminary annotation result. Additionally, to improve the accuracy and completeness of the annotation, if a second-highest value λ₂ exists, the cell type corresponding to λ₂ is added to the original annotation, providing a more refined annotation hierarchy. However, in certain cases, if the λ value is 0 or negative, its biological significance is unclear, so it is not included in the final annotation results. Through the use of this optimization strategy, λ₁ and λ₂ are calculated according to Formulas (7) and (8), and the final cell annotation results are generated based on these values (Figure 3). In Table 3, the similarity score matrix for clusters 0 to 5 is presented. The highest similarity score for cluster 1 is 0.00119, while for cluster 4, it is 0.000532. The full similarity score matrix is available in File S3. The annotation results of FPCAM are presented in File S4. Each cell cluster is annotated as one or two cell types if it meets the criteria.

### 3.4. Results of Model Evaluation and Comparison

We also employed three state-of-the-art cell annotation tools—SCSA, SciBet, and SingleR—to annotate the same dataset. The annotation results from these tools were integrated using the Boyer–Moore majority voting algorithm to generate a pseudo cell type as a reference standard for model evaluation. As shown in Table 4, both FPCAM and SCSA achieved an accuracy of 89.7%, outperforming the other models. The SingleR model, using ImmGenData and MouseRNAseqData as reference files, achieved accuracies of 72.4% and 48.3%, respectively. The SciBet model, trained on the 20mouse_organs and Lung Smart-seq2 datasets, achieved accuracies of 75.9% and 51.7%, respectively. To further assess the stability of models that rely on different reference annotation files, we calculated their *R* values. SingleR showed an *R* of 0.241, and SciBet had an *R* of 0.242, indicating lower stability and a stronger dependency on reference data. In contrast, SCSA had an *R* of 0, reflecting consistent performance. Overall, both FPCAM and SCSA demonstrated higher accuracy and stability, highlighting their advantages in cell annotation tasks. Notably, FPCAM also showed the ability to identify cell subtypes. Detailed annotation results for all models are provided in File S5.

### 3.5. Construction of the Online Data Analysis Platform FPCAM

**Figure 4:**
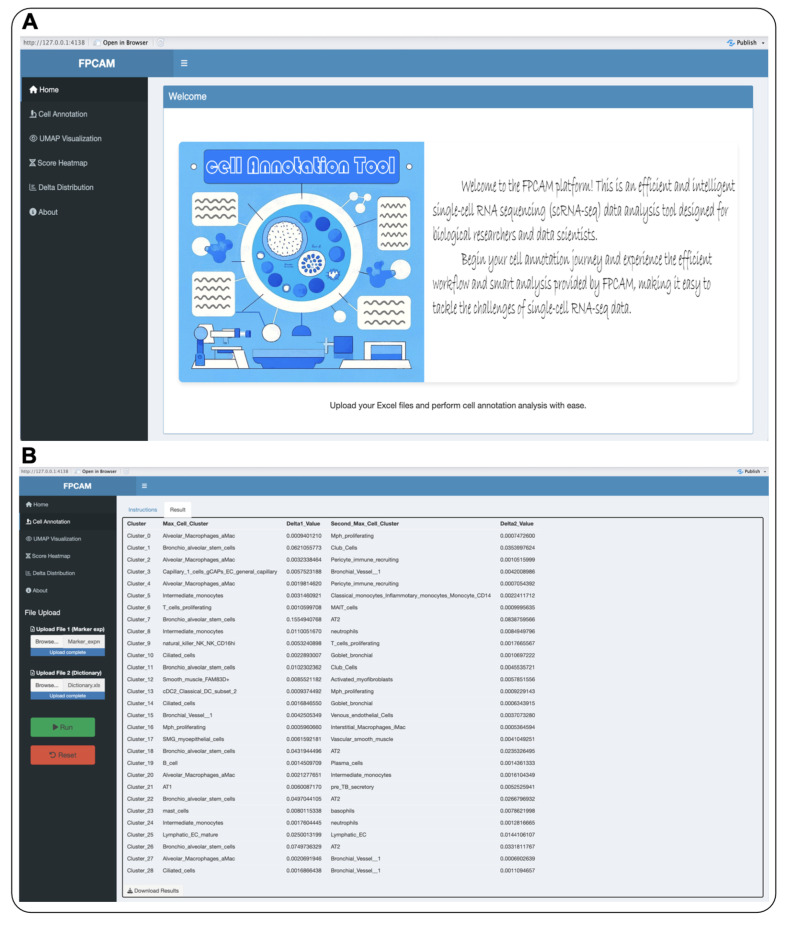
FPCAM online analysis platform. (**A**)**.** The left-side menu bar offers modules like “Cell Annotation”, “UMAP Visualization”, “Score Heatmap”, and “Delta Distribution”, guiding users through data upload and processing. (**B**) Annotation results of FPCAM based on the GSE135893 dataset.

FPCAM provides an integrated user interface designed to streamline the processing and analysis of scRNA-seq data. The menu bar on the left side of the interface includes functional modules such as “Cell Annotation”, “UMAP Visualization”, “Score Heatmap”, and “Delta Distribution”. Users can select appropriate modules based on their research needs, and the platform will guide them through data upload and processing steps via an intuitive interface. Within each module, users are first required to upload data files that meet the platform’s format requirements, typically in Excel or H5 format for single-cell data. Once the files are uploaded, users can initiate the analysis process by simply clicking the “Run” button (Figure 4A). We demonstrated the annotation results using the human pulmonary fibrosis dataset GSE135893, which showed that FPCAM is capable of accurately predicting cell subtypes (Figure 4B).

### 3.6. Visualization and Interactive Features

**Figure 5:**
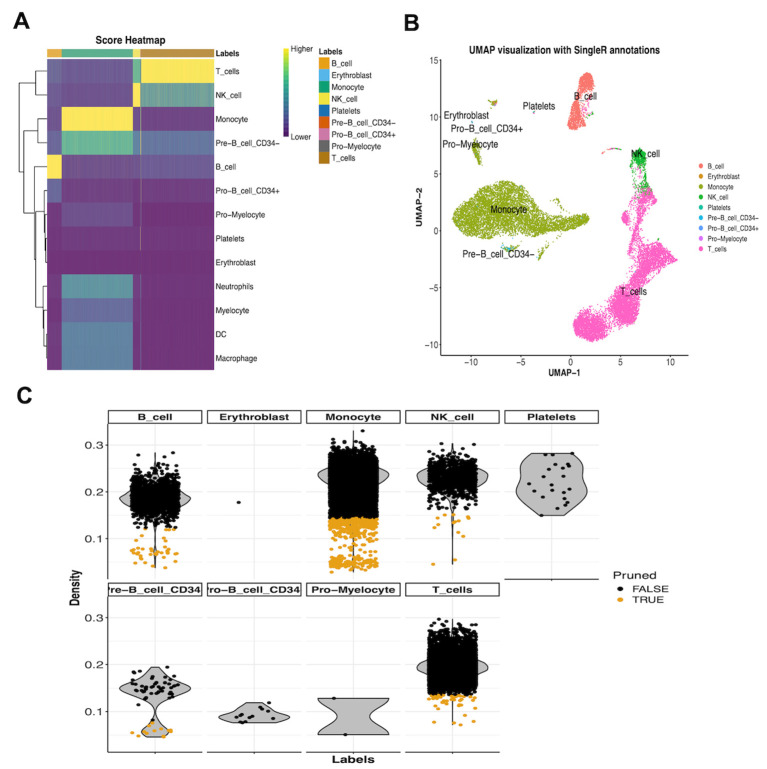
FPCAM visualization features. (**A**) The score heatmap from FPCAM illustrates the similarity between cell annotation results and the reference dataset. (**B**) The UMAP plot visualizes the spatial distribution of cell populations using the 20k_PBMC_3p_HT_nextgem_Chromium_X_filtered_feature_bc_matrix dataset. (**C**) The Delta distribution plot highlights differences between cell types based on the same test dataset. Users can download analysis results, including images and tables, for further exploration.

Additionally, the workflow covers several key steps such as data quality control, dimensionality reduction, normalization, and clustering to ensure the accuracy and interpretability of the data. The result display section provides real-time analytical feedback, allowing users to observe the analysis results through visual charts. As shown in Figure 5A, the score heatmap calculated by FPCAM demonstrates the degree of similarity between the cell annotation results and the reference dataset. In Figure 5B, the UMAP plot generated by FPCAM shows the spatial distribution of cell populations, using the 20k_PBMC_3p_HT_nextgem_Chromium_X_filtered_feature_bc_matrix test dataset as an example. Figure 5C presents the Delta distribution plot created by FPCAM, reflecting the differences between cell types, also using the same test dataset (20k_PBMC_3p_HT_nextgem_Chromium_X_filtered_feature_bc_matrix). FPCAM analysis results, including images and tables, can be saved via the download button on the page for further analysis by users. Finally, we compiled a list of human and mouse pulmonary fibrosis scRNA-Seq datasets through a literature review. These datasets are in relatively standard formats and can be integrated with the FPCAM tool for pulmonary fibrosis-related data analysis (Table 5).

## 4. Discussion

Accurate cell-type annotation is critically important in single-cell transcriptome data analysis. However, the complexity of disease-specific data and the high heterogeneity of cell populations present significant challenges to the annotation process. To address these challenges, we developed FPCAM, a pulmonary fibrosis-specific model and fully automated cell annotation tool built on the R Shiny platform. This tool integrates existing marker gene databases with advanced computational methods, providing an efficient, precise, and user-friendly solution for pulmonary fibrosis cell annotation. FPCAM not only offers a comprehensive cell annotation workflow but also supports downstream analyses, presents results through interactive visualizations, and includes data download capabilities. The development of this tool provides strong support for the efficient processing and in-depth analysis of single-cell RNA sequencing data.

Currently, research on the sequelae of COVID-19 remains in its early stages, with pulmonary fibrosis considered one of the key issues [41]. In patients with severe disease, fibrosis-related cytokines such as Transforming Growth Factor (TGF-β), Tumor Necrosis Factor (TNF-α), and Interleukin (IL)-6 are significantly elevated in peripheral blood, which is closely associated with the development of pulmonary fibrosis [42]. Among them, TGF-β can induce the transformation of alveolar fibroblasts into myofibroblasts, leading to the abnormal deposition of the extracellular matrix (ECM), resulting in tissue damage and immune cell infiltration, thereby driving the fibrosis process [43]. Additionally, the abnormal activation of M1 and M2 macrophages is also considered a key factor in the occurrence and development of pulmonary fibrosis [44]. In recent years, studies have revealed that some long non-coding RNAs (LncRNAs) play an important role in the pathogenesis of pulmonary fibrosis. For example, DACH1 (LncRNA) can inhibit the activation of lung fibroblasts and the excessive deposition of the ECM by binding to SRSF1 and suppressing the expression of SRSF1 and CTNNB1 proteins, which helps alleviate pulmonary fibrosis [45]. With the rapid development of single-cell sequencing technology, research on pulmonary fibrosis has gradually advanced to the single-cell level. However, as cell annotation is a critical step in single-cell data analysis, existing annotation tools are still limited, and the related technologies are not yet mature. There is an urgent need to develop more precise and efficient annotation methods to enhance the analytical capabilities of pulmonary fibrosis research.

Cell annotation is a core component of scRNA-seq data analysis, directly affecting the reliability and depth of downstream biological research. In recent years, although significant progress has been made in cell annotation techniques based on scRNA-seq, several challenges remain. Multiple cell annotation methods have been proposed, including similarity-based methods like SingleR [12], machine learning-based methods like SciBet [21], integration-based strategies like SCSA [20], and deep learning-based methods like scAnnotate [46]. These methods have demonstrated certain advantages in specific applications but also have limitations. Firstly, many existing cell annotation tools heavily rely on pre-built reference databases. For example, SingleR mainly uses datasets like ImmGenData and BlueprintEncode for cell-type matching. However, when the target dataset significantly differs from the reference dataset in terms of origin, annotation accuracy can significantly decrease, particularly when studying rare cell types or in specific disease contexts [17]. scCATCH, which combines manual and automated annotation techniques, shows certain advantages in identifying cell subtypes, but there are still areas that need improvement, such as marker gene selection, cell subtype construction, and the standardization of evaluation systems [47]. Secondly, machine learning and deep learning methods (such as SciBet [21] and scAnnotate [46]) can predict cell types in an unsupervised environment. However, their performance depends on high-quality training data and requires substantial known cell marker information. The generalization capability of the models is often limited by the diversity and quality of the training data, making them difficult to adapt to complex biological contexts [48]. Additionally, the “black-box” nature of deep learning models limits their biological interpretability, making it challenging to intuitively reveal underlying biological mechanisms [49]. Moreover, most current cell annotation tools are primarily based on healthy tissues or common sample data, with a lack of cell annotation models tailored for specific disease contexts. For instance, pulmonary fibrosis is a highly heterogeneous disease, and the cellular composition of its pathological tissues may vary significantly due to individual differences. Existing cell annotation methods often struggle to accurately distinguish specific cell subpopulations in the pulmonary fibrosis microenvironment, such as fibroblasts at different differentiation stages or the degree of immune cell infiltration. Therefore, developing cell annotation models specifically for pulmonary fibrosis is of great significance for a deeper understanding of its pathological mechanisms and for advancing precision medicine.

FPCAM, the model proposed in this study, employs a weighted dictionary construction strategy, integrating marker gene data from multiple authoritative sources and optimizing them for the specific disease context of pulmonary fibrosis. This model not only significantly improves the accuracy of cell annotation but also demonstrates stronger adaptability in identifying disease-specific cell types. Additionally, the FPCAM online tool features an intuitive visualization interface, allowing users to flexibly adjust parameters and easily apply it across different datasets. FPCAM is compatible with mainstream single-cell transcriptome analysis software (e.g., Seurat and FindMarkers), enabling users to manually adjust cell marker genes and cell cluster information based on custom-built dictionaries to meet different research needs [28]. In terms of performance evaluation, we conducted an in-depth comparison of FPCAM with other mainstream cell annotation tools, such as SCSA, SingleR, and SciBet, by using a voting mechanism. The experimental results demonstrated that FPCAM achieved an accuracy of 89.7%, significantly outperforming other models, further validating its advantages and reliability in pulmonary fibrosis research.

Although FPCAM demonstrates high accuracy and stability in the single-cell annotation task for pulmonary fibrosis, there are still certain limitations and areas for improvement. First, FPCAM relies on a predefined cell–gene association dictionary, the completeness and accuracy of which directly affect the annotation results. For rare or under-represented cell types, the existing dictionary may not provide comprehensive coverage, leading to annotation biases or omissions. In addition, significant challenges remain in distinguishing closely related cell subtypes and cellular states. Therefore, future research should further expand and optimize the marker gene database and integrate public database APIs for real-time dynamic updates to enhance the model’s adaptability and accuracy. Second, although FPCAM uses a voting mechanism to reduce bias from individual tools, different algorithms may still perform differently on specific datasets. Future versions could consider incorporating machine learning or deep learning methods to enable the model to adjust weights adaptively based on training data, thereby improving its generalization ability and enhancing its applicability in complex data environments. Additionally, FPCAM currently focuses primarily on cell types related to pulmonary fibrosis. It could be extended to other disease areas in the future, such as cancer and neurodegenerative diseases, to enhance its applicability in a broader range of biological research contexts and meet a wider variety of research needs. Finally, there is still room for optimization in user experience and computational efficiency. While the Shiny platform provides a user-friendly interface, computational performance could be improved when processing large-scale datasets. Future versions could consider incorporating cloud computing or GPU acceleration technologies to improve data processing speed and further optimize the user interface, making FPCAM more accessible to researchers without a bioinformatics background, thus increasing its practicality and potential for widespread use.

In a nutshell, this study developed a cell annotation method for pulmonary fibrosis scRNA-Seq data—FPCAM. The platform integrates preprocessing, cell annotation, and data visualization functionalities for single-cell transcriptome data, aiming to improve annotation accuracy and adaptability. As an efficient and flexible cell annotation tool, FPCAM provides novel technical means and research approaches for pulmonary fibrosis and other single-cell transcriptome studies. It holds the potential to play an important role in related disease research and precision medicine.

## 5. Conclusions

Given the diversity and complexity of cellular profiles across different disease contexts, generic annotation methods often fall short of practical needs, underscoring the importance of developing disease-specific annotation models. In this study, we introduce FPCAM, a fully automated cell annotation tool tailored for pulmonary fibrosis. By integrating a high-quality marker gene database with advanced computational strategies, FPCAM significantly improves annotation accuracy and robustness. Achieving an accuracy of 89.7%, it outperforms existing mainstream tools while reducing dependency on reference datasets. FPCAM offers a reliable approach to cell annotation in pulmonary fibrosis and holds promise for supporting the advancement of precision medicine.

## Figures and Tables

**Table 1:** References for the pulmonary fibrosis-specific cell–gene dictionary.

*Species*	*Cell Identities*	*Citations*	*PMID*	*References*
Human	61	380	PMID: 37291214	Sikkema et al., *Nature Medicine*, 2023 [5]
Human	80	123	PMID: 36543915	Madissoon et al., *Nature Genetics*, 2023 [6]
Human and Mouse	27	121	PMID: 34936882	Sun et al., *Developmental Cell*, 2022 [7]
Human	58	1416	PMID: 33208946	Travaglini et al., *Nature*, 2020 [8]
Mouse	22	387	PMID: 30318149	Cohen et al., *Cell*, 2018 [9]
Mouse	7	1206	PMID: 30069044	Montoro et al., *Nature*, 2018 [30]

**Table 2:** Display of basal resting cells from the dictionary file.

*Cell Cluster*	*Marker Gene*	*Number of Votes Received*	*Votes*	*Percent*
Basal resting	TP63	4	21	4/21
Basal resting	KRT5	4	21	4/21
Basal resting	KRT15	3	21	1/7
Basal resting	FXYD3	2	21	2/21
Basal resting	KRT14	2	21	2/21
Basal resting	KRT17	2	21	2/21
Basal resting	EPCAM	1	21	1/21
Basal resting	ELF3	1	21	1/21
Basal resting	IGFBP2	1	21	1/21
Basal resting	SERPINF1	1	21	1/21

**Table 3:** Similarity score matrix based on marker genes and pulmonary fibrosis annotation dictionary.

*Cells/Clusters*	*Cluster 0*	*Cluster 1*	*Cluster 2*	*Cluster 3*	*Cluster 4*	*Cluster 5*
AT1	0	0	0	5.89 × 10^−5^	1.80 × 10^−5^	0
AT2	0	0	0	3.34 × 10^−5^	2.04 × 10^−5^	0
AT2_proliferating	0	0	0	5.56 × 10^−5^	3.40 × 10^−5^	0
Activated_myofibroblasts	0	0	1.34 × 10^−5^	0	0	0.002383
Airway_smooth_muscle	0	0	0	0	4.63 × 10^−5^	7.79 × 10^−6^
Alveolar_Macrophages_aMac	0	0.000543	0.00017	0.000272	0.000532	6.92 × 10^−6^
Alveolar_fibroblasts_1_Lipofibroblasts	0	0	0	0	0	0.002304
Alveolar_fibroblasts_2_Matrix_fibroblast_2_Adventitial_fibroblasts	0	3.15 × 10^−5^	0	0	0	0.002601
Arterial_endothelial_Cells	0	1.28 × 10^−5^	0	0	0	1.91 × 10^−5^
B_cell	0.00119	0.000356	0.00016	0.000256	0	0

**Table 4:** Evaluation of model annotation performance.

*Model*	*Reference File*	*Unannotated*	*Cell Subtype*	*Accuracy*
SCSA	CellMarker	No	No	0.897
SCSA	PanglaoDB	No	No	0.897
SingleR	ImmGendate	No	No	0.724
SingleR	MouseRNAseqData	Yes	Yes	0.483
SciBet	Tabula Muris [20 mouse organs]	No	Yes	0.759
SciBet	Tabula Muris [Lung Smart seq2]	No	No	0.517
FPCAM	FP Dictionary	No	Yes	0.897

Note: *R*(SCSA) = 0; *R*(SingleR) = 0.241; *R*(SciBet) = 0.242

**Table 5:** List of human and mouse pulmonary fibrosis scRNA-seq datasets.

*Dataset*	*Samples*	*Citations*	*PMID*	*References*
GSE132771	24	551	PMID: 32317643	Tsukui, Tatsuya et al. *Nature Communications*. 2020 [31]
GSE141259	60	556	PMID: 32678092	Strunz, Maximilian et al. *Nature Communications*. 2020 [32]
GSE267904	24	21	PMID: 38951642	Franzén, Lovisa et al. *Nature Genetics*. 2024 [33]
GSE104154	6	15	PMID: 34108218	Xie, Ting et al. *Science Advances*. 2021 [34]
GSE267861	2	0	PMID: 39587675	Pan, Caizhe et al. *Journal of Translational Medicine*. 2024 [35]
GSE135893	34	873	PMID: 32832598	Habermann, Arun C et al. *Science Advances*. 2020 [36]
GSE122960	17	1177	PMID: 30554520	Reyfman, Paul A et al. *American Journal of Respiratory and Critical Care Medicine*. 2019 [37]
GSE136831	78	1108	PMID: 32832599	Adams, Taylor S et al. *Science Advances*. 2020 [38]
GSE128033	18	619	PMID: 31221805	Morse, Christina et al. *The European Respiratory Journal*. 2019 [39]
GSE86618	540	8	PMID: 27942595	Xu, Yan et al. *JCI Insight*. 2016 [40]
GSE94555	6	8	PMID: 27942595	Xu, Yan et al. *JCI Insight*. 2016 [40]

## Data Availability

The computational data generated in this study and the source code of the FPCAM model are freely available at https://github.com/guojunliu7/FPCAM (accessed on 24 April 2025).

## Supplementary Materials

The following supporting information can be downloaded at: https://www.mdpi.com/article/10.3390/biology14050479/s1, File S1: Marker genes and expression levels across different clusters; File S2: Construction of the pulmonary fibrosis dictionary file ; File S3: Similarity score matrix; File S4: Annotation results of the FPCAM model; File S5: Comparison of annotation results across all models; Table S1: Statistics of single cell sequencing results; Table S2: Statistics of alignment result; Table S3: Quantitative analysis of single cell sequencing data. Figure S1: Statistics of up-regulated marker genes in all samples. X axis represents the cluster of clustering plan, Y axis represents the relative number of marker genes.

## Abbreviations

Single-cell RNA sequencing (scRNA-seq); Human Lung Cell Atlas (HLCA); Fibrosis Pulmonary Cell Type Annotation Model (FPCAM); unique molecular identifier (UMI); range (R); accuracy (Acc).

## References

[1] Zhang S., Li X., Lin J., Lin Q., Wong K.-C. (2023). Review of single-cell RNA-seq data clustering for cell-type identification and characterization. RNA.

[2] Wen L., Li G., Huang T., Geng W., Pei H., Yang J., Zhu M., Zhang P., Hou R., Tian G. (2022). Single-cell technologies: From research to application. Innovation.

[3] Chen G., Ning B., Shi T. (2019). Single-cell RNA-seq technologies and related computational data analysis. Front. Genet..

[4] Huynh T., Cang Z. (2024). Topological and geometric analysis of cell states in single-cell transcriptomic data. Brief. Bioinform..

[5] Sikkema L., Ramírez-Suástegui C., Strobl D.C., Gillett T.E., Zappia L., Madissoon E., Markov N.S., Zaragosi L.-E., Ji Y., Ansari M. (2023). An integrated cell atlas of the lung in health and disease. Nat. Med..

[6] Madissoon E., Oliver A.J., Kleshchevnikov V., Wilbrey-Clark A., Polanski K., Richoz N., Orsi A.R., Mamanova L., Bolt L., Elmentaite R. (2023). A spatially resolved atlas of the human lung characterizes a gland-associated immune niche. Nat. Genet..

[7] Sun X., Perl A.K., Li R., Bell S.M., Sajti E., Kalinichenko V.V., Kalin T.V., Misra R.S., Deshmukh H., Clair G. (2022). A census of the lung: CellCards from LungMAP. Dev. Cell.

[8] Travaglini K.J., Nabhan A.N., Penland L., Sinha R., Gillich A., Sit R.V., Chang S., Conley S.D., Mori Y., Seita J. (2020). A molecular cell atlas of the human lung from single-cell RNA sequencing. Nature.

[9] Cohen M., Giladi A., Gorki A.D., Sinha R., Gillich A., Sit R.V., Chang S., Conley S.D., Mori Y., Seita J. (2018). Lung single-cell signaling interaction map reveals basophil role in macrophage imprinting. Cell.

[10] Zhang A.W., O’Flanagan C., Chavez E.A., Lim J.L.P., Ceglia N., McPherson A., Wiens M., Walters P., Chan T., Hewitson B. (2019). Probabilistic cell-type assignment of single-cell RNA-seq for tumor microenvironment profiling. Nat. Methods.

[11] Zhang Z., Luo D., Zhong X., Choi J.H., Ma Y., Wang S., Mahrt E., Guo W., Stawiski E.W., Modrusan Z. (2019). SCINA: A semi-supervised subtyping algorithm of single cells and bulk samples. Genes.

[12] Aran D., Looney A.P., Liu L., Wu E., Fong V., Hsu A., Chak S., Naikawadi R.P., Wolters P.J., Abate A.R. (2019). Reference-based analysis of lung single-cell sequencing reveals a transitional profibrotic macrophage. Nat. Immunol..

[13] Kiselev V.Y., Yiu A., Hemberg M. (2018). scmap: Projection of single-cell RNA-seq data across data sets. Nat. Methods.

[14] Tan Y., Cahan P. (2019). SingleCellNet: A computational tool to classify single cell RNA-seq data across platforms and across species. Cell Syst..

[15] Alquicira-Hernandez J., Sathe A., Ji H.P., Nguyen Q., Powell J.E. (2019). scPred: Accurate supervised method for cell-type classification from single-cell RNA-seq data. Genome Biol..

[16] Clarke Z.A., Andrews T.S., Atif J., Pouyabahar D., Innes B.T., MacParland S.A., Bader G.D. (2021). Tutorial: Guidelines for annotating single-cell transcriptomic maps using automated and manual methods. Nat. Protoc..

[17] Abdelaal T., Michielsen L., Cats D., Hoogduin D., Mei H., Reinders M.J.T., Mahfouz A. (2019). A comparison of automatic cell identification methods for single-cell RNA sequencing data. Genome Biol..

[18] Domínguez Conde C., Xu C., Jarvis L.B., Rainbow D.B., Wells S.B., Gomes T., Howlett S.K., Suchanek O., Polanski K., King H.W. (2022). Cross-tissue immune cell analysis reveals tissue-specific features in humans. Science.

[19] Ma F., Pellegrini M. (2020). ACTINN: Automated identification of cell types in single cell RNA sequencing. Bioinformatics.

[20] Cao Y., Wang X., Peng G. (2020). SCSA: A cell type annotation tool for single-cell RNA-seq data. Front. Genet..

[21] Li C., Liu B., Kang B., Liu Z., Liu Y., Chen C., Ren X., Zhang Z. (2020). SciBet as a portable and fast single cell type identifier. Nat. Commun..

[22] Brown J., Pirrung M., McCue L.A. (2017). FQC dashboard: Integrates FastQC results into a web-based, interactive, and extensible FASTQ quality control tool. Bioinformatics.

[23] Bolger A.M., Lohse M., Usadel B. (2014). Trimmomatic: A flexible trimmer for illumina sequence data. Bioinformatics.

[24] Martin M. (2011). Cutadapt removes adapter sequences from high-throughput sequencing reads. Embnet. J..

[25] Dobin A., Davis C.A., Schlesinger F., Drenkow J., Zaleski C., Jha S., Batut P., Chaisson M., Gingeras T.R. (2013). STAR: Ultrafast universal RNA-seq aligner. Bioinformatics.

[26] Liao Y., Smyth G.K., Shi W. (2014). featureCounts: An efficient general purpose program for assigning sequence reads to genomic features. Bioinformatics.

[27] Abrams Z.B., Johnson T.S., Huang K., Payne P.R.O., Coombes K. (2019). A protocol to evaluate RNA sequencing normalization methods. BMC Bioinform..

[28] Satija R., Farrell J.A., Gennert D., Schier A.F., Regev A. (2015). Spatial reconstruction of single-cell gene expression data. Nat. Biotechnol..

[29] Jia L., Yao W., Jiang Y., Li Y., Wang Z., Li H., Huang F., Li J., Chen T., Zhang H. (2022). Development of interactive biological web applications with R/shiny. Brief. Bioinform..

[30] Montoro D.T., Haber A.L., Biton M., Vinarsky V., Lin B., Birket S.E., Yuan F., Chen S., Leung H.M., Villoria J. (2018). A revised airway epithelial hierarchy includes CFTR-expressing ionocytes. Nature.

[31] Tsukui T., Sun K.H., Wetter J.B., Wilson-Kanamori J.R., Hazelwood L.A., Henderson N.C., Adams T.S., Schupp J.C., Poli S.D., Rosas I.O. (2020). Collagen-producing lung cell atlas identifies multiple subsets with distinct localization and relevance to fibrosis. Nat. Commun..

[32] Strunz M., Simon L.M., Ansari M., Kathiriya J.J., Angelidis I., Mayr C.H., Tsidiridis G., Lange M., Mattner L.F., Yee M. (2020). Alveolar regeneration through a Krt8+ transitional stem cell state that persists in human lung fibrosis. Nat. Commun..

[33] Franzén L., Olsson Lindvall M., Hühn M., Ptasinski V., Setyo L., Keith B.P., Collin A., Oag S., Volckaert T., Borde A. (2024). Mapping spatially resolved transcriptomes in human and mouse pulmonary fibrosis. Nat. Genet..

[34] Xie T., Kulur V., Liu N., Deng N., Wang Y., Rowan S.C., Yao C., Huang G., Liu X., Taghavifar F. (2021). Mesenchymal growth hormone receptor deficiency leads to failure of alveolar progenitor cell function and severe pulmonary fibrosis. Sci. Adv..

[35] Pan C., Wei H., Chen B., Wu L., Song J., Zhang Q., Wu X., Liang G., Chen W., Wang Y. (2024). Inhalation of itraconazole mitigates bleomycin-induced lung fibrosis via regulating SPP1 and C3 signaling pathway pivotal in the interaction between phagocytic macrophages and diseased fibroblasts. J. Transl. Med..

[36] Habermann A.C., Gutierrez A.J., Bui L.T., Yahn S.L., Winters N.I., Calvi C.L., Peter L., Chung M.I., Taylor C.J., Jetter C. (2020). Single-cell RNA sequencing reveals profibrotic roles of distinct epithelial and mesenchymal lineages in pulmonary fibrosis. Sci. Adv..

[37] Reyfman P.A., Walter J.M., Joshi N., Anekalla K.R., McQuattie-Pimentel A.C., Chiu S., Fernandez R., Akbarpour M., Chen C.I., Ren Z. (2019). Single-Cell Transcriptomic Analysis of Human Lung Provides Insights into the Pathobiology of Pulmonary Fibrosis. Am. J. Respir. Crit. Care. Med..

[38] Adams T.S., Schupp J.C., Poli S., Ayaub E.A., Neumark N., Ahangari F., Chu S.G., Raby B.A., DeIuliis G., Januszyk M. (2020). Single-cell RNA-seq reveals ectopic and aberrant lung-resident cell populations in idiopathic pulmonary fibrosis. Sci. Adv..

[39] Morse C., Tabib T., Sembrat J., Buschur K.L., Bittar H.T., Valenzi E., Jiang Y., Kass D.J., Gibson K., Chen W. (2019). Proliferating SPP1/MERTK-expressing macrophages in idiopathic pulmonary fibrosis. Eur. Respir. J..

[40] Xu Y., Mizuno T., Sridharan A., Du Y., Guo M., Tang J., Wikenheiser-Brokamp K.A., Perl A.T., Funari V.A., Gokey J.J. (2016). Single-cell RNA sequencing identifies diverse roles of epithelial cells in idiopathic pulmonary fibrosis. JCI. Insight..

[41] Nalbandian A., Sehgal K., Gupta A., Madhavan M.V., McGroder C., Stevens J.S., Cook J.R., Nordvig A.S., Shalev D., Sehrawat T.S. (2021). Post-acute COVID-19 syndrome. Nat. Med..

[42] King C.S., Mannem H., Kukreja J., Aryal S., Tang D., Singer J.P., Bharat A., Behr J., Nathan S.D. (2022). Lung transplantation for patients with COVID-19. Chest.

[43] Ojo A.S., Balogun S.A., Williams O.T., Ojo O.S. (2020). Pulmonary fibrosis in COVID-19 survivors: Predictive factors and risk reduction strategies. Pulm. Med..

[44] Sun Y., Xu H., Lu T., Li T., Wang Y., Fan X., Jiang Y., Cai M., He P., Liu J. (2023). Progress in understanding the role and therapeutic targets of polarized subtypes of macrophages in pulmonary fibrosis. Cell Biochem. Biophys..

[45] Sun J., Jin T., Niu Z., Guo J., Guo Y., Yang R., Wang Q., Gao H., Zhang Y., Li T. (2022). LncRNA DACH1 protects against pulmonary fibrosis by binding to SRSF1 to suppress CTNNB1 accumulation. Acta Pharm. Sin. B.

[46] Ji X., Tsao D., Bai K., Tsao M., Xing L., Zhang X. (2023). scAnnotate: An automated cell-type annotation tool for single-cell RNA-sequencing data. Bioinform. Adv..

[47] Shao X., Liao J., Lu X., Xue R., Ai N., Fan X. (2020). scCATCH: Automatic annotation on cell types of clusters from single-cell RNA sequencing data. iScience.

[48] Gundogdu P., Alamo I., Nepomuceno-Chamorro I.A., Dopazo J., Loucera C. (2023). SigPrimedNet: A Signaling-Informed Neural Network for scRNA-seq Annotation of Known and Unknown Cell Types. Biology.

[49] Xu C., Jackson S.A. (2019). complex and complex biological data. Genome Biol..

