# Supplementary figures and images for: FPCAM: A Weighted Dictionary-Driven Model for Single-Cell Annotation in Pulmonary Fibrosis
Source: Biology (Basel). 2025 Apr 26;14(5):479. doi: 10.3390/biology14050479 (PMC12108865; doi:10.3390/biology14050479)

# Statistics of marker genes

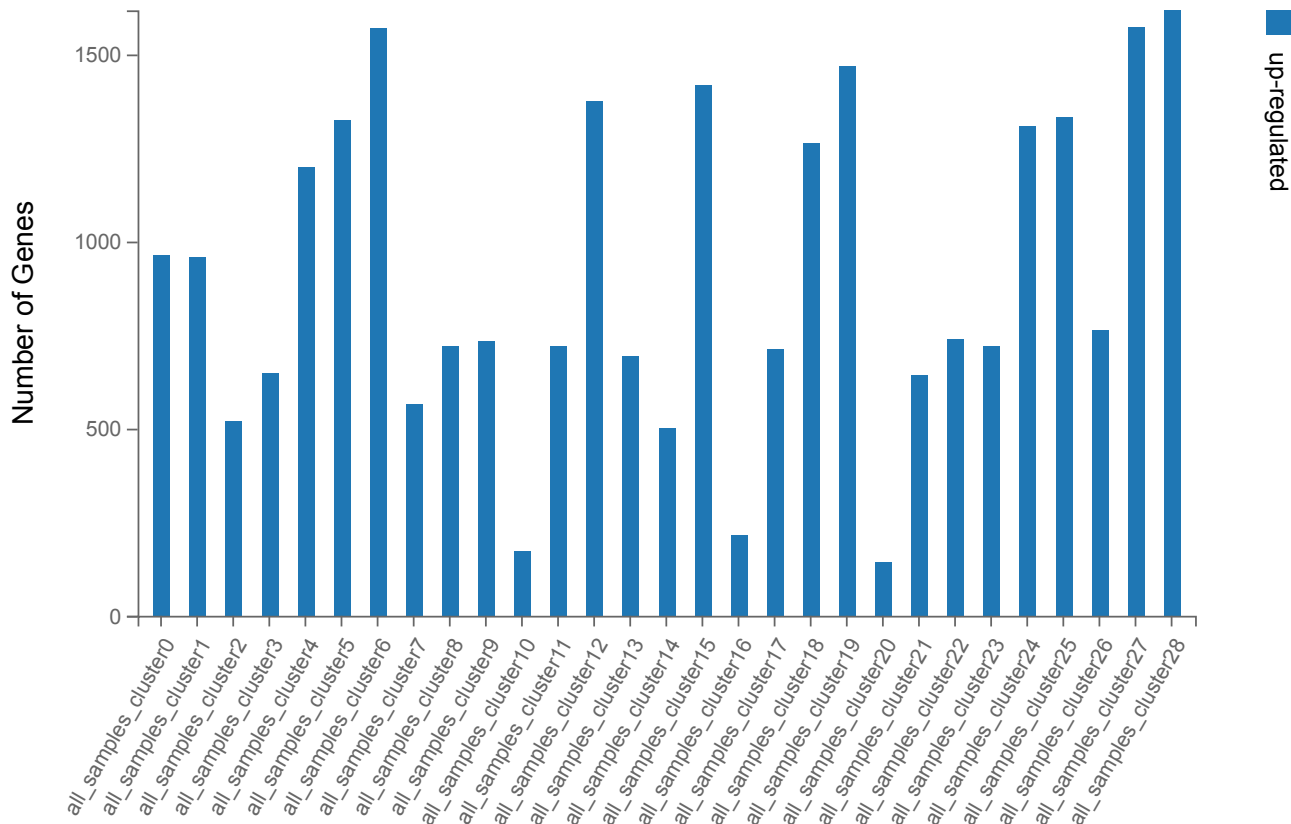

Supplement: Supplementary file 1 [file biology-14-00479-s001.zip › Figure_S1.pdf]
